# Cerium oxide nanoparticles attenuate acute kidney injury induced by intra-abdominal infection in Sprague–Dawley rats

**DOI:** 10.1186/s12951-015-0135-z

**Published:** 2015-10-24

**Authors:** Nandini D. P. K. Manne, Ravikumar Arvapalli, Niraj Nepal, Tolou Shokuhfar, Kevin M. Rice, Shinichi Asano, Eric R. Blough

**Affiliations:** Center for Diagnostic Nanosystems, Marshall University, Huntington, WV USA; Department of Public Health, Marshall University, Huntington, WV USA; Department of Pharmaceutical Sciences and Research, Marshall University, Huntington, WV USA; Department of Pharmacology, Physiology and Toxicology, Marshall University, Huntington, WV USA; Department of Mechanical Engineering and Engineering Mechanics, Michigan Technological University, Houghton, MI USA

**Keywords:** Cerium oxide nanoparticles, Polymicrobial insult, Acute kidney injury, Reactive oxygen species, Peritonitis

## Abstract

**Background:**

Intra-abdominal infection or peritonitis is a cause for great concern due to high mortality rates. The prognosis of severe intra-abdominal infection is significantly diminished in the presence of acute kidney injury (AKI) which is often characterized by renal tubular cell death that can lead to renal failure. The purpose of the current study is to examine the therapeutic efficacy of cerium oxide (CeO_2_) nanoparticles for the treatment of peritonitis-induced AKI by polymicrobial insult.

**Results:**

A one-time administration of CeO_2_ nanoparticles (0.5 mg/kg) in the absence of antibiotics or other supportive care, attenuated peritonitis-induced tubular dilatation and the loss of brush border in male Sprague–Dawley rats. These improvements in renal structure were accompanied by decreases in serum cystatin-C levels, reduced renal oxidative stress, diminished Stat-3 phosphorylation and an attenuation of caspase-3 cleavage suggesting that the nanoparticle treatment improved renal glomerular filtration rate, diminished renal inflammation and reduced renal apoptosis. Consistent with these data, further analysis demonstrated that the CeO_2_ nanoparticle treatment diminished peritonitis-induced increases in serum kidney injury molecule-1 (KIM-1), osteopontin, β-2 microglobulin and vascular endothelial growth factor-A (VEGF-A) levels. In addition, the nanoparticle attenuated peritonitis-induced hyperglycemia along with increases in blood urea nitrogen (BUN), serum potassium and sodium.

**Conclusion:**

CeO_2_ nanoparticles scavenge reactive oxygen species and attenuate polymicrobial insult induced increase in inflammatory mediators and subsequent AKI. Taken together, the data indicate that CeO_2_ nanoparticles may be useful as an alternative therapeutic agent or in conjunction with standard medical care for the treatment of peritonitis induced acute kidney injury.

## Background

Intra-abdominal infection-induced peritonitis is characterized by an overwhelming immune response in reaction to the presence of infectious agents that can lead to multi-organ failure and death if not properly managed. Acute kidney injury (AKI) induced by polymicrobial insult is a cause of great concern as it increases mortality rates and is also associated with increased financial burden and length of hospital stay [1]. Despite advances in medical science and care, sepsis still baffles the research community due to its complex pathophysiology and rapid progression making current therapeutic interventions ineffective.

Intra-abdominal infection-induced acute renal failure is caused by a multitude of factors that includes components of ischemia–reperfusion injury, hypoperfusion, endothelial dysfunction, coagulation defects, cytokine storm and apoptosis [2–4]. Most studies to-date point that the single most cause for intra-abdominal infection induced AKI is the development of the systemic inflammatory response syndrome (SIRS) which is mediated by increased oxidative stress [5, 6]. Other studies have shown that intra-abdominal infection causes injury to renal tubular cells by damaging the endothelium that leads to increased permeability [7]. These changes are histologically evident by loss of brush border and increased tubular dilatation [8]. At the cellular level, it is presumed that polymicrobial insult causes tubular cell death by caspase mediated apoptosis [2]. As such therapeutic interventions directed towards elimination of reactive oxygen species (ROS) may be warranted for the treatment of AKI.

Recent developments in nanotechnology have given rise to several promising compounds that can effectively scavenge ROS [9–11]. Among those, CeO_2_ nanoparticles have been increasingly used in biomedical field for the treatment of various pathologies involving oxidative stress [12, 13] and inflammation [14, 15]. A property that is unique to CeO_2_ nanoparticles is the ability to cycle between Ce^+3^ (reduced) and Ce^+4^ (oxidized) states which allows them to effectively scavenge ROS [16]. Other studies have shown that CeO_2_ nanoparticles behave as a superoxide dismutase [17] and catalase [18] mimetic depending on the redox state of the particles. Researches have exploited this property of CeO_2_ nanoparticles for the treatment of diabetes [19, 20], cancer [21, 22] and ischemic stroke [23].

Previously we have shown that CeO_2_ nanoparticles inhibit intra-abdominal infection induced liver and heart dysfunction [24]. Whether CeO_2_ nanoparticles can be used to attenuate polymicrobial insult-induced AKI is not known. In continuation with our previous study, we evaluated the therapeutic efficacy of CeO_2_ nanoparticles for the treatment of polymicrobial insult-induced AKI by examining the effect of nanoparticle intervention on renal histological damage, oxidative stress, inflammation, apoptosis and changes in circulating AKI biomarkers.

## Results

### Characterization of nanoceria

**Fig. 1:**
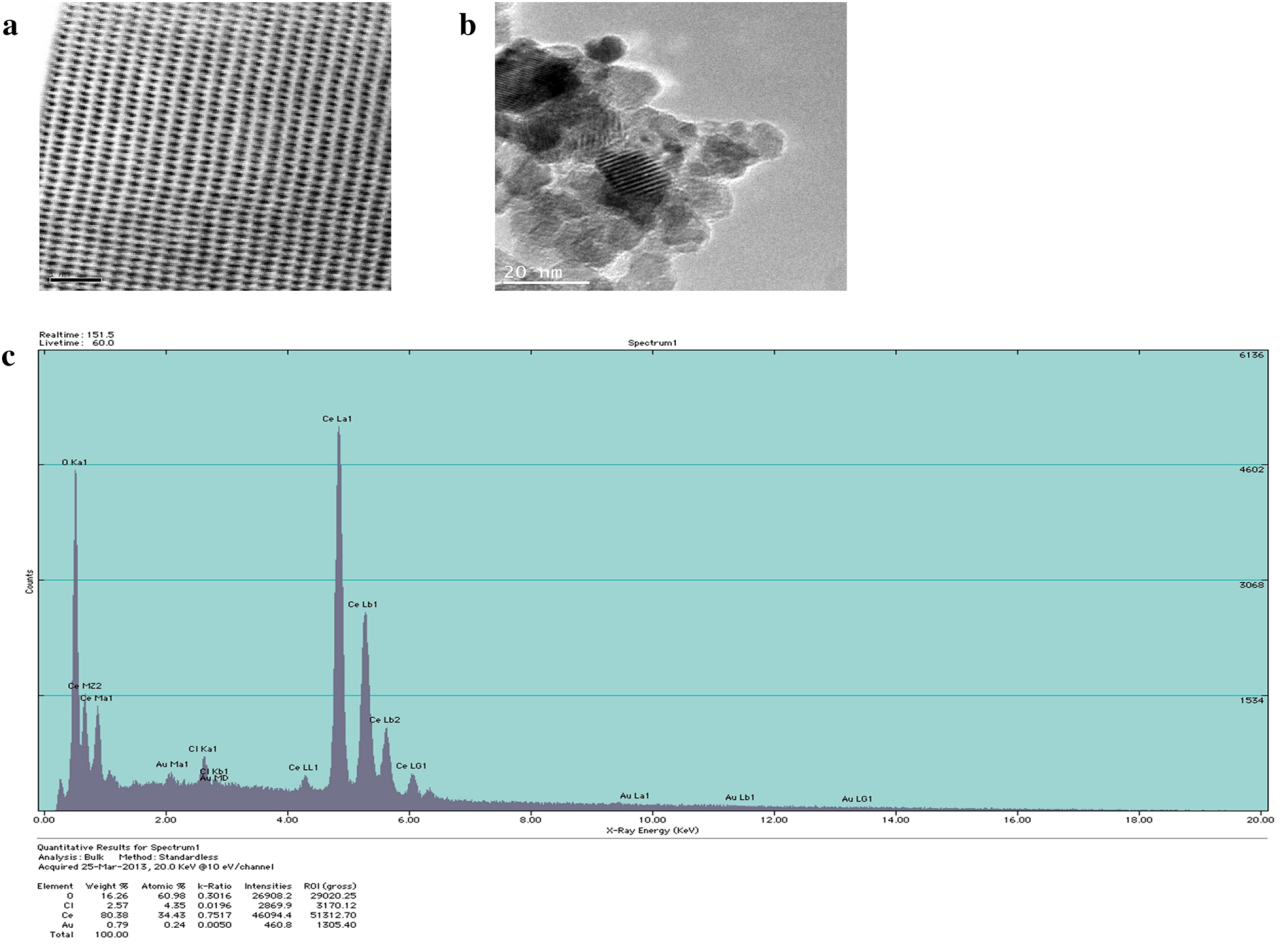
Characterization of CeO_2_ nanoparticles. **a** Scanning transmission electron microscopy (STEM), **b** Transmission electron microscopy (TEM), **c** Energy-Dispersive X-Ray Spectroscopy (EDS)

Scanning transmission electron microscopy (STEM) was used to demonstrate the ordered structure of nanoparticles while transmission electron microscopy (TEM) demonstrated the size of CeO_2_ nanoparticles to be in between 10-40 nm (Fig. 1a, b). Energy dispersive X-ray spectroscopy (EDS) indicated that the content of cerium and oxygen in the sample to be 80.38 and 16.26 %, respectively (Fig. 1c).

### Cerium oxide nanoparticles attenuate peritonitis induced renal damage and breakdown of tubular F-actin

**Fig. 2:**
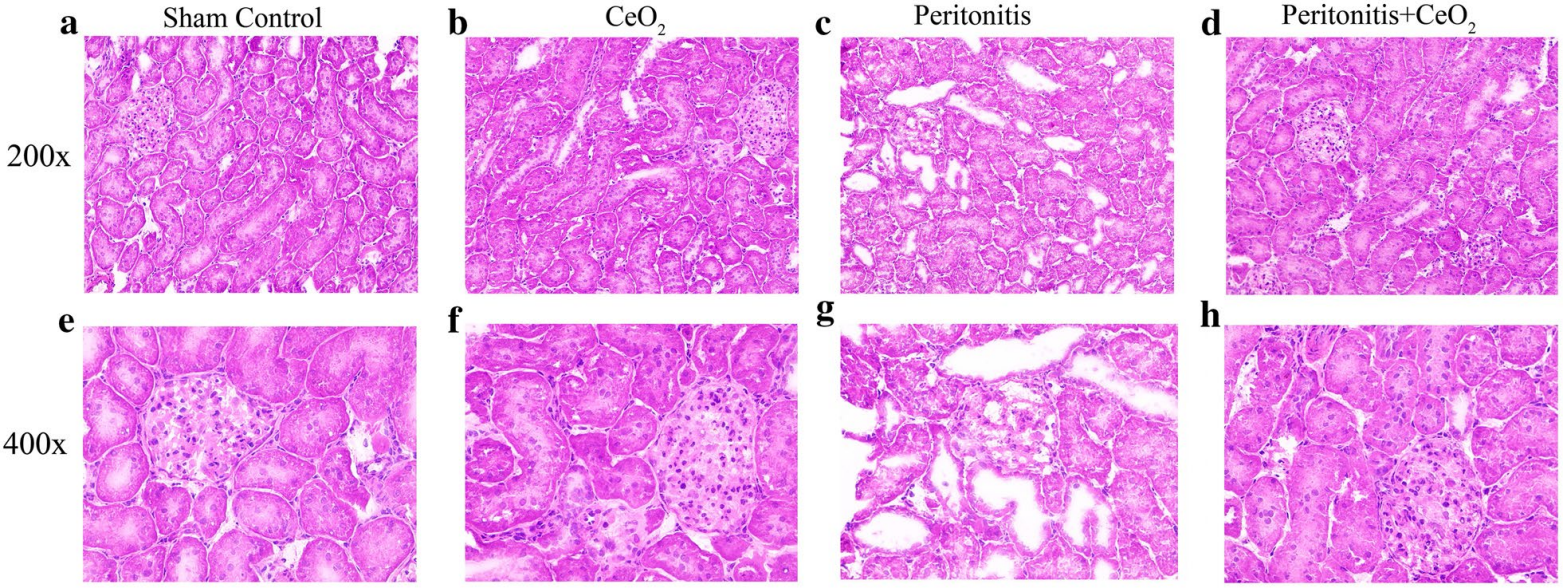
CeO_2_ nanoparticles attenuate peritonitis induced renal damage. Hematoxylin and eosin staining of kidney sections 18 h after polymicrobial insult. **a** Control, **b** CeO_2_, **c** Peritonitis, **d** Peritonitis + CeO_2_ (200× magnification), **e** Control, **f** CeO_2_, **g** Peritonitis, **h** Peritonitis + CeO_2_ (400× magnification) (n = 4/group)

**Fig. 3:**
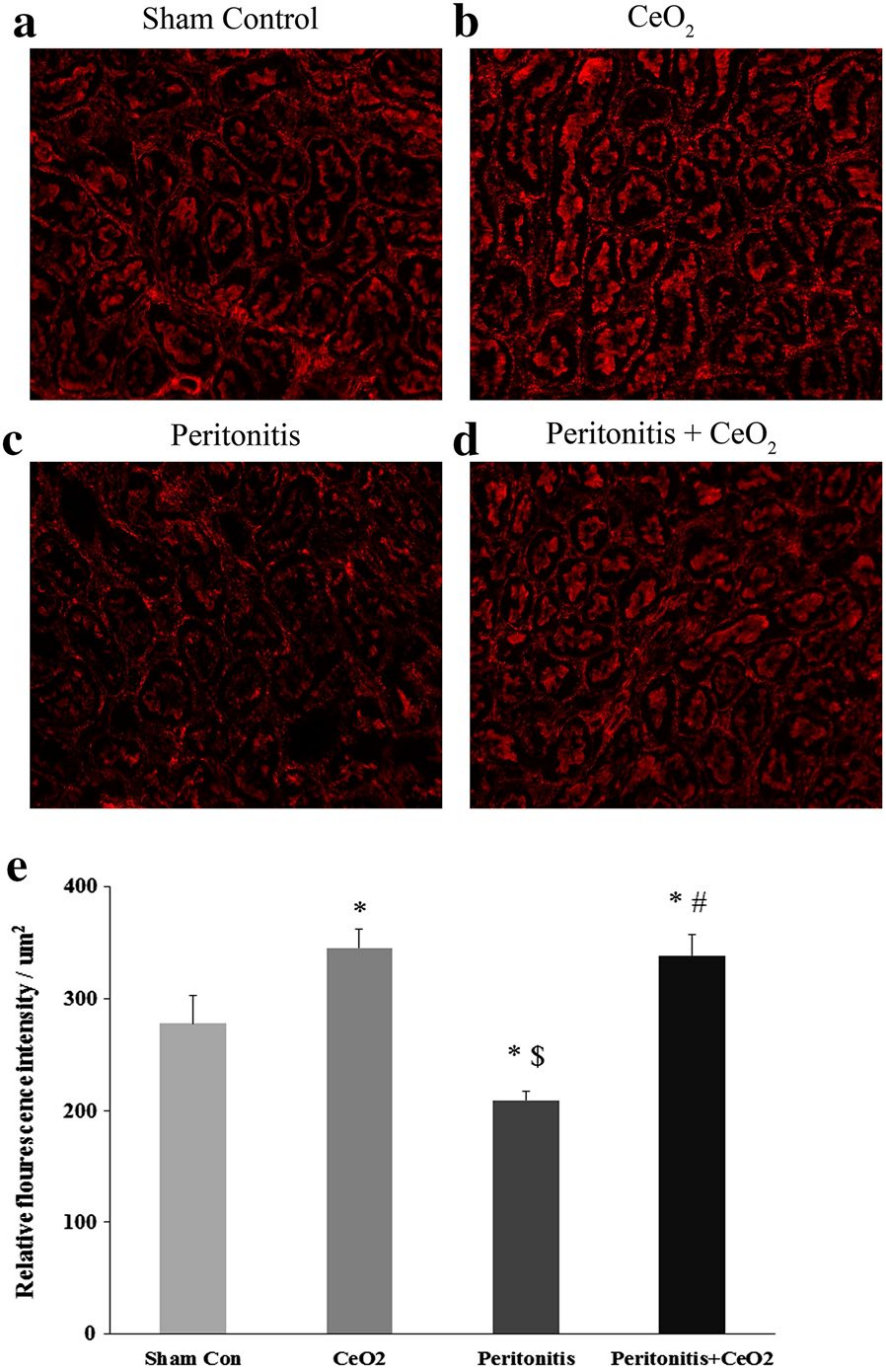
CeO_2_ nanoparticles attenuate peritonitis induced loss of F-actin. Rhodamine phalloidin staining of F-actin of kidney sections 18 h after polymicrobial insult. **a** Control, **b** CeO_2_, **c** Peritonitis, **d** Peritonitis + CeO_2_ and **e** Relative fluorescence intensity as a measure of F-actin levels. 200× magnification. *P < 0.05 compared to control group, ^$^ *P* < 0.05 compared to CeO_2_ group and ^#^ *P* < 0.05 compared to peritonitis group (n = 3/group)

Polymicrobial insult-induced AKI was characterized by renal tubular dilatation, loss of brush border and damage to the glomerular capillary network (Fig. 2c, g) which appeared to be attenuated with nanoparticle treatment (Fig. 2d, h). Renal sections of peritonitis induced animals showed marked loss of F-actin in proximal tubular cells (Fig. 3c) which was attenuated with nanoparticle treatment (Fig. 3d). The mean fluorescence intensity for F-actin was higher in peritonitis +CeO_2_ group by 61 % when compared to peritonitis group alone (Fig. 3e) (*P* < 0.05).

### Cerium oxide nanoparticles attenuate peritonitis induced oxidative stress, stat-3 activation, cleavage of caspase 3 and serum biomarkers of renal failure

**Fig. 4:**
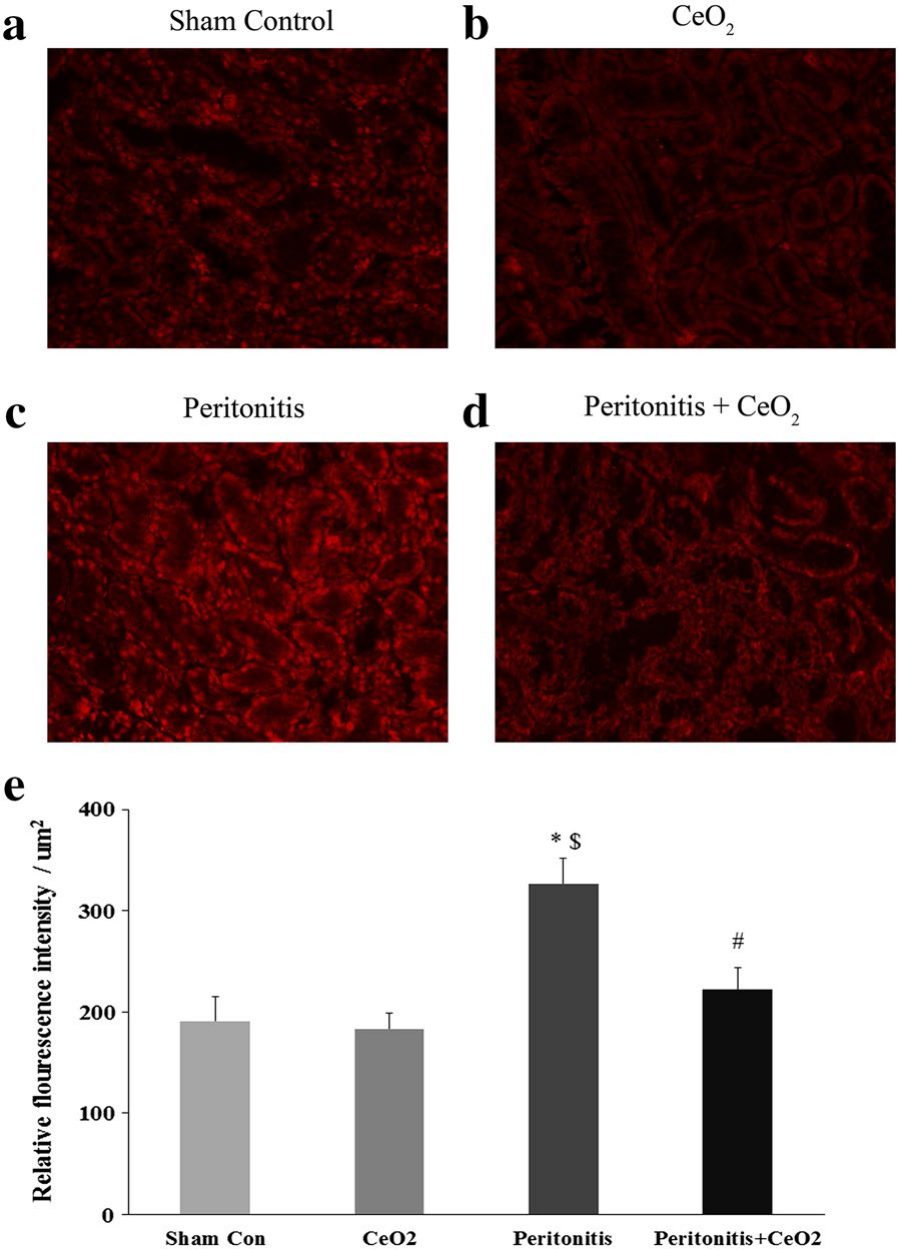
CeO_2_ nanoparticles attenuate peritonitis induced increase in renal superoxide levels. Dihydroethidium staining of kidney sections at 18 h after polymicrobial insult. **a** Control, **b** CeO_2_, **c** Peritonitis, **d** Peritonitis + CeO_2_ and **e** Quantification of superoxide levels in different groups. 200× magnification. *P < 0.05 compared to control group, ^$^ *P* < 0.05 compared to CeO_2_ group and ^#^ *P* < 0.05 compared to peritonitis group (n = 4/group)

**Fig. 5:**
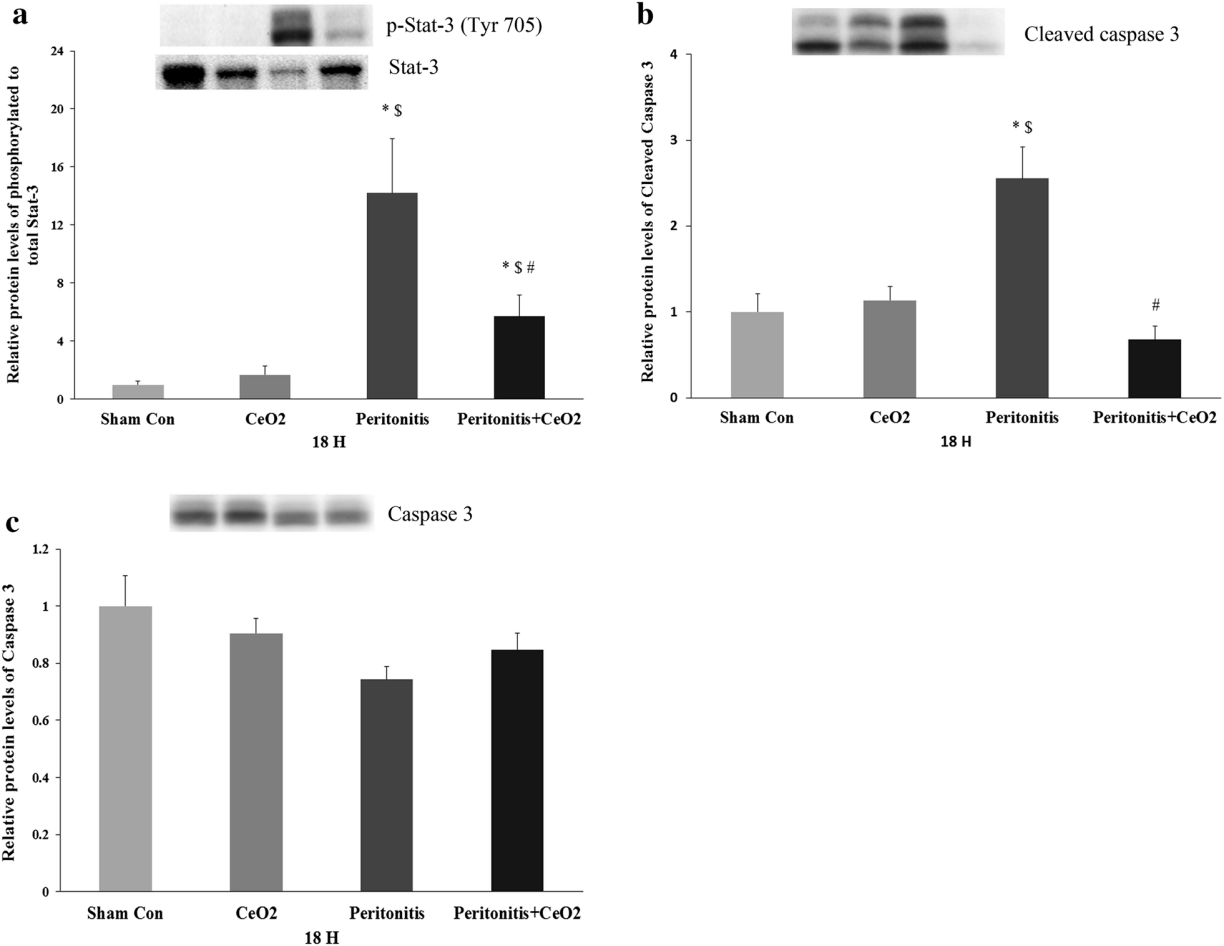
CeO_2_ nanoparticles attenuate peritonitis induced renal inflammation and apoptosis. **a** Levels of phosphorylated to total Stat-3. **b** Relative levels of cleaved caspase-3 and **c** Relative levels of caspase-3 as determined by immunoblotting. *P < 0.05 compared to control group, ^$^ *P* < 0.05 compared to CeO_2_ group and ^#^ *P* < 0.05 compared to peritonitis group (n = 5–6/group)

Compared to control and treated animals, the renal sections obtained from animals suffering from peritonitis exhibited increased superoxide levels (Fig. 4c, e; *P* < 0.05). Similarly, nanoparticle treatment decreased peritonitis induced increases in the ratio of phosphorylated to total levels of Stat-3 (Fig. 5a; *P* < 0.05) and caspase-3 cleavage (Fig. 5b; *P* < 0.05) at 18 h. Compared to the control group and treated animals, peritonitis increased the levels of β-2 microglobulin at 3 h and 18 h and the levels of KIM-1, cystatin-C, osteopontin and VEGF-A at 18 h (Table 1; *P* < 0.05).

**Table 1:** CeO_2_ nanoparticles attenuate peritonitis induced increase in biomarkers of AKI

Analyte	Sham control 3 h	CeO_2_ 3 h	Peritonitis 3 h	Peritonitis + CeO_2_ 3 h	Sham control 18 h	CeO_2_ 18 h	Peritonitis 18 h	Peritonitis + CeO_2_ 18 h
β2-Microglobulin (µg/mL)	54.00 ± 3.00	49.67 ± 2.40	76.00 ± 1.53^*$^	77.33 ± 1.76^*$^	52.00 ± 3.51	32.67 ± 1.45^*^	128.00 ± 5.85^*$^	78.33 ± 7.21^*$#^
Cystatin-C (ng/mL)	578.67 ± 14.25	633.00 ± 30.57	686.00 ± 14.47	690.33 ± 42.10	699.67 ± 52.67	555.33 ± 41.63	1890.00 ± 62.45^*$^	1093.33 ± 37.56^*$#^
KIM-1 (ng/mL)	Below LLOQ	Below LLOQ	Below LLOQ	Below LLOQ	Below LLOQ	Below LLOQ	3.27 ± 0.13	2.50 ± 0.10^#^
Osteopontin (ng/mL)	10.80 ± 1.20	11.00 ± 0.58	11.00 ± 0.58	12.33 ± 0.33	9.70 ± 0.00	7.93 ± 0.23*	72.33 ± 4.84^*$^	36.33 ± 1.76^*$#^
VEGF-A (pg/mL)	Below LLOQ	Below LLOQ	Below LLOQ	Below LLOQ	Below LLOQ	Below LLOQ	459.00 ± 12.70	296.67 ± 31.33^#^

*LLOQ* lower limit of quantitation

*P < 0.05 compared to control group, ^$^ *P* < 0.05 compared to CeO_2_ group and ^#^ *P* < 0.05 compared to peritonitis group (n = 6/group)

### Cerium oxide nanoparticles attenuate peritonitis induced changes in serum biochemical parameters

Peritonitis caused a decrease in serum levels of sodium at 3 h and an increase in levels of potassium at 18 h (Table 2; *P* < 0.05). Nanoparticle treatment attenuated peritonitis-induced changes in serum sodium and potassium (Table 2; *P* < 0.05). Similarly, peritonitis induced early hyperglycemia and increases in levels of BUN at 3 h time point were attenuated with nanoparticles treatment (Table 2; *P* < 0.05).

**Table 2:** CeO_2_ nanoparticles attenuate peritonitis insult induced alterations in serum biochemical parameters

Analyte	Sham control 3 h	CeO_2_ 3 h	Peritonitis 3 h	Peritonitis + CeO_2_ 3 h	Sham control 18 h	CeO_2_ 18 h	Peritonitis 18 h	Peritonitis + CeO_2_ 18 h
Glucose (mg/dL)	284.00 ± 8.11	262.29 ± 10.13	426.88 ± 19.74^*$^	308.57 ± 22.58^#^	245.33 ± 13.25	290.63 ± 17.16^*^	105.50 ± 8.53^*$^	125.25 ± 5.06^*$^
Blood urea nitrogen (mg/dL)	22.00 ± 1.10	21.00 ± 1.00	29.88 ± 1.97^*$^	24.71 ± 1.17^#^	19.17 ± 0.54	18.50 ± 0.89	71.30 ± 7.20^*$^	57.37 ± 3.09^$^
Sodium (mmol/L)	142.43 ± 0.72	142.86 ± 0.88	136.75 ± 1.36^*$^	140.14 ± 0.51^#^	142.00 ± 1.21	141.63 ± 0.91	138.80 ± 0.84	140.63 ± 0.50
Potassium (mmol/L)	5.91 ± 0.37	5.44 ± 0.11	6.36 ± 0.27	6.30 ± 0.23	5.62 ± 0.24	5.75 ± 0.33	7.83 ± 0.24^*$^	7.06 ± 0.21^*$#^

*P < 0.05 compared to control group, ^$^ *P* < 0.05 compared to CeO_2_ group and ^#^ *P* < 0.05 compared to peritonitis group (n = 6/group)

## Discussion

Intra-abdominal infection or peritonitis-induced AKI has a poor prognosis despite recent advances in medical care. Studies have shown that peritonitis-induced AKI is associated with increases in oxidative and nitrosative stress which promote renal inflammation, tubular dilatation, vacuolization, sloughing of epithelial cells and loss of brush border which if severe can cause renal failure [25, 26]. CeO_2_ nanoparticles have been shown to act as an anti-oxidant and anti-inflammatory agent in the treatment of several diseases including cancer and diabetes [27, 28]. Similarly, previous work by our laboratory has showed that CeO_2_ nanoparticles can attenuate intra-abdominal infection-induced animal death along with liver and heart damage [24]. With this in mind, we hypothesized that CeO_2_ nanoparticles could be used to prevent peritonitis induced AKI.

Previous studies have shown that peritonitis induced by polymicrobial insult leads to marked tubular damage along with loss of brush border and derangement in glomerular capillary network [29]. We found that the CeO_2_ nanoparticle treatment attenuated peritonitis-induced damage to the renal glomeruli and tubules (Fig. 1). AKI is characterized by the loss of F-actin that leads to disruption in cytoskeleton network and impairs renal structural and functional integrity [30]. To address this possibility, we next sought to determine whether the nanoparticles can prevent peritonitis-induced changes in renal structural integrity. As expected we found that peritonitis caused a significant decrease F-actin associated fluorescence which appeared to be attenuated with CeO_2_ nanoparticle treatment (Fig. 3). Taken together, these data suggest that the nanoparticle treatment was associated with diminished AKI.

It is thought that peritonitis is characterized by increases in oxidative stress which can result in an uncontrolled systemic inflammatory response and multi-organ failure [31]. Studies have shown that CeO_2_ nanoparticles are potent ROS scavengers and that they can act as catalase and SOD mimetics [32]. Supporting these data, we found that CeO_2_ nanoparticle treatment significantly attenuated peritonitis induced-increases in renal superoxide oxide levels (Fig. 4). To extend these findings, we next examined the phosphorylation (activation) of the inflammatory mediated Jak-Stat proteins [33]. The Jak-Stat pathway has been shown to participate in the development of diabetic nephropathy, renal fibrosis and ischemia reperfusion injury where it functions as a key regulator of cytokine and growth factor signaling [34]. Additional data has also demonstrated that Stat-3 signaling is necessary for the hypoxia-induced transcription of VEGF [35]. Here we found that peritonitis-induced the activation of Stat-3 and increased serum VEGF levels. (Fig. 5a; Table 1). Similarly, a growing body of evidence suggests that the apoptosis of renal tubular cells is one of the major causes for AKI in peritonitis induced by polymicrobial insult [36]. Other work has demonstrated that the cleavage of caspase-3 activates gelsolin which has F-actin severing properties [37]. The loss of F-actin in turn, causes cytoskeletal derangement that can result in apoptotic cell death [30]. In agreement with our fluorescent measurement of actin levels, we found that peritonitis induced-cleavage of caspase-3 was significantly attenuated by CeO_2_ nanoparticle treatment (Figs. 3, 5a). Taken together and consistent with our histological findings of diminished renal damage, these data suggest that nanoparticle treatment is associated with decreased renal superoxide levels, reduced renal inflammation, and diminished renal apoptosis.

In addition to changes in cellular structure, AKI has also been shown to be associated with decreases in glomerular filtration rate and alterations in serum KIM-1, β-2 microglobulin, and osteopontin levels [38–40]. As expected, peritonitis was associated with alterations in the serum level of all of these variables which appeared to be significantly diminished with nanoparticle treatment (Table 1). In a similar fashion, hyponatremia, hyperkalemia, hyperglycemia, and increased blood urea nitrogen levels are generally found in acute renal failure with decreased renal tubular flow rate [8, 41]. Consistent with these findings, we found similar changes in the present study, and importantly, demonstrated a reduction in these variables with nanoparticle treatment (Table 2).

## Conclusion

**Fig. 6:**
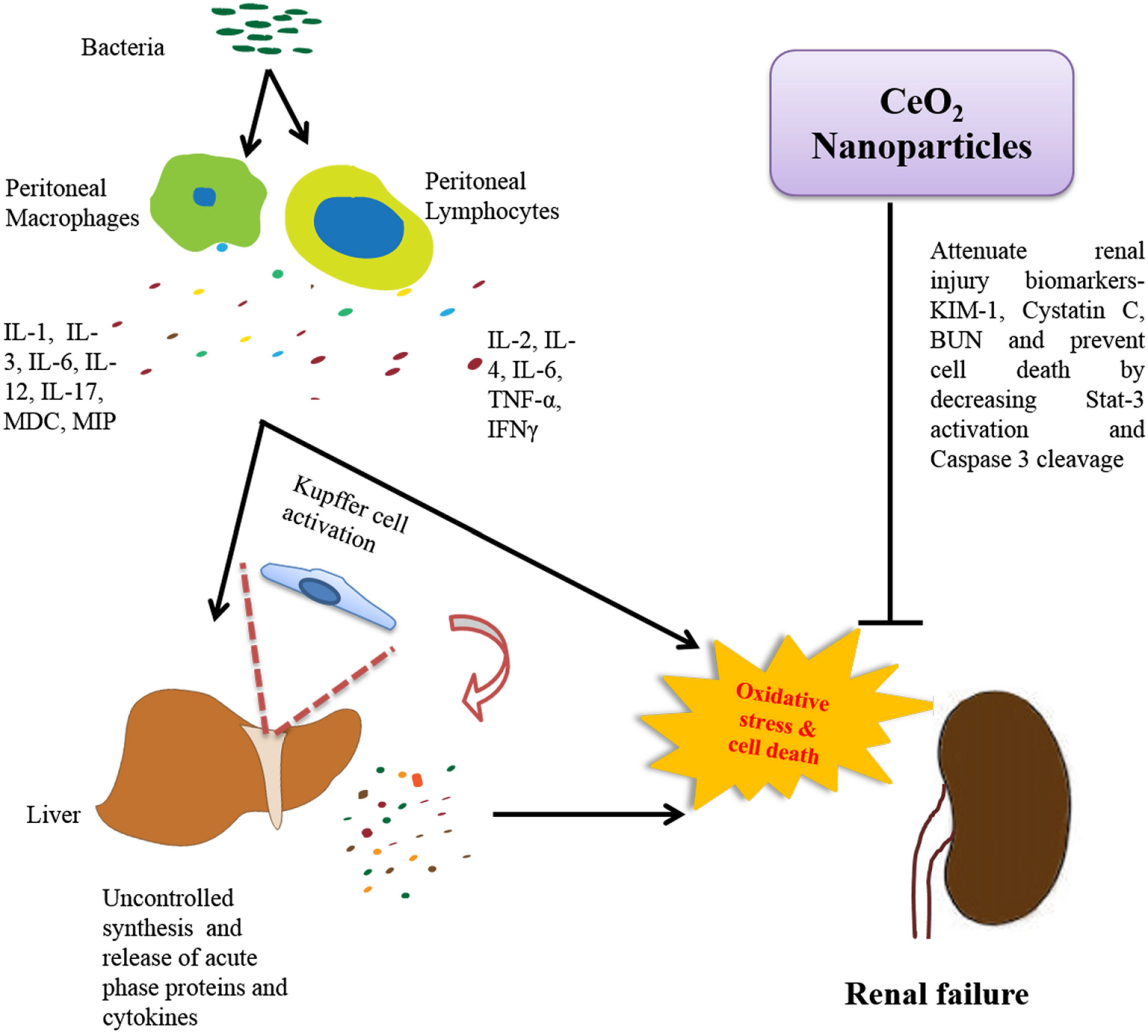
Schematic representation of mechanism of action of CeO_2_ nanoparticles on polymicrobial insult induced AKI

The results of the current study suggest that a single, “stand-alone” dose of CeO_2_ nanoparticles confers protection against severe polymicrobial insult-induced acute kidney injury. Our data suggest that the CeO_2_ nanoparticles act by scavenging reactive oxygen species which is associated with diminished caspase-3 activation, reduced loss of F-actin and less damage to the tubules. Improvements in kidney structure were accompanied by evidence of increased renal function and decreased serum biomarker levels of renal injury (Fig. 6). Taken together, these data suggest that further investigation into the potential utility of using CeO_2_ nanoparticles for the treatment of polymicrobial-induced AKI may be warranted.

## Methods

### Characterization of CeO_2_ nanoparticles

CeO_2_ nanoparticles were commercially purchased from US Research Nanomaterials Inc. (Houston, TX, USA). Scanning transmission electron microscopy (STEM) images were obtained by using Aberration Corrected Analytical Electron Microscope (TEM/STEM JEOL JEM-ARM200CF, Japan) operated at 200 keV. Particle size was obtained using JEOL JEM-2010 transmission electron microscope (TEM). Sample purity was estimated by energy dispersive X-ray spectroscopy using Noran Voyager EDX software.

### Polymicrobial insult and CeO_2_ nanoparticle treatment

Male Sprague–Dawley rats aged 10 weeks were purchased from Hill-Top laboratories and allowed to acclimatize for 2 weeks prior to experimentation. All surgical procedures were performed in accordance with the guidelines provided by Marshall University Institutional Animal Care and Use Committee (IACUC), and Association for Assessment and Accreditation of Laboratory Animal Care (AAALAC). Briefly, animals were anesthetized under isoflurane and a small mid ventral incision of 0.5 cm was made. Sham controls and CeO_2_ only groups were injected with 5 ml/kg of 5 % sterile dextrose solution intra-peritoneally (*i.p*) while peritonitis and peritonitis +CeO_2_ groups received cecal inoculum of 600 mg/kg BW in 5 ml/kg BW of 5 % sterile dextrose solution *i.p* as described previously [42]. Cecal material was obtained from healthy donor rats. The sham control and peritonitis groups were injected with 200 µl of sterile distilled water intravenously while the CeO_2_ and peritonitis +CeO_2_ groups received CeO_2_ nanoparticles (0.5 mg/kg) in 200 µl of sterile distilled water via tail vein injection.

### Tissue collection

Animals were humanely sacrificed under anesthesia and the kidneys were excised, capsule removed and washed in Krebs–Ringer bicarbonate buffer (KRB) to remove any blood. Kidneys were frozen in liquid nitrogen and stored at −80 °C until further analysis. Serum was obtained from whole blood by centrifugation at 5000×*g* for 10 min at room temperature and stored at −80 °C.

### Renal histology and staining for F-actin

Frozen kidneys were sectioned (4 µm) with a Leica CM1950 cryostat and transferred to poly-l-lysine coated slides. Hematoxylin and eosin staining was performed using Histoperfect kit (BBC Biochemical, Seattle WA, USA) to assess kidney morphology and imaged using Evos XL microscope (Life Technologies, Grand Island, NY, USA).

Renal sections were stained for F-actin using rhodamine phalloidin (Life technologies, Grand Island, NY, USA). Briefly, frozen sections were washed with PBS and fixed in 4 % methanol free formaldehyde for 10 min and washed with PBS (3 × 5 min). Sections were then permeabilized with 0.3 % Triton X-100 in PBS for 20 min and washed with PBS (3 × 5 min). After blocking with 1 % BSA for 30 min, the tissue sections were incubated with 0.165 µM rhodamine phalloidin for 20 min in the dark, washed with PBS (3 × 5 min), cover slipped and then imaged with a Evos FL microscope (Life Technologies, Grand Island, NY, USA). Four images per section from at least three different animals from each group were evaluated for fluorescence intensity using Image J analysis software.

### Estimation of renal superoxide levels

Superoxide levels in renal sections were estimated using dihydroethidium staining as described previously [43]. Briefly, sections were washed with PBS and incubated with 5 mM dihydroethidium for 1 h at room temperature in the dark. After washing with PBS (3 × 5 min), sections were imaged with an Evos FL microscope (Life Technologies, Grand Island, NY, USA). Four images per section from at least four different animals from each group were evaluated for fluorescence intensity using Image J analysis software.

### SDS-PAGE and immunoblotting

Approximately 100 mg of frozen kidney was taken and pulverized to a fine powder, suspended in 900 µl of T-PER (Pierce, Rockford, IL, USA) containing 1 % protease and phosphatase inhibitors and homogenized. Supernants were collected by centrifugation (10,000×*g* for 10 min at 4 °C and the protein concentration determined using the 660 nm assay (Pierce, Rockford, IL, USA). Samples were equally diluted with 4× Laemilli buffer and then subjected to electrophoresis on 10 % PAGEr Gold Precast gel (Lonza, Rockland, ME, USA) before transfer to nitrocellulose membranes as detailed elsewhere [44]. Equal loading of protein was verified by Ponceau S staining of nitrocellulose membranes. Membranes were blocked with 5 % milk in TBST for 1 h and later probed with primary antibodies against p-stat-3 (Tyr 705), stat-3, cleaved caspase-3 and caspase-3 (Cell Signaling Technology, Danvers, MA, USA). After exposure to the primary antibodies, membranes were washed with TBST (3 × 5 min) and incubated with secondary anti-rabbit (Cell Signaling Technology, Danvers, MA, USA) for 1 h at room temperature. Immunoreactivity was visualized using Supersignal West Pico Chemiluminiscent substrate (Pierce, Rockford, IL, USA) and quantified by Fluorchem 9900 software (Protein Simple, Santa Clara, CA, USA).

### Multiplex immunoassay and serum biochemical analysis

Serum samples from different animals in each group were pooled and sent to Myriad RBM (Austin, TX, USA) for the analysis of KIM-1, cystatin-C, osteopontin, β-2 microglobulin and VEGF-A using the rodent kidney MAP. Samples were run in triplicate for statistical analysis. The amount of glucose, BUN, sodium and potassium were determined in serum using an Abaxis VetScan^®^ analyzer (Abaxis, Union City, CA, USA).

### Statistical analysis

Results are presented in the form of mean ± standard error of mean. Differences in dependent variables across groups were determined by one way analysis of variance (ANOVA) with Student Newman Keul’s post hoc analysis or ANOVA on ranks with Student Newman Keul’s post hoc analysis for non-normally distributed samples. Where appropriate a *t* test was used. A probability value of *P* < 0.05 was accepted to be statistically significant.
